# Plasma extracellular vesicle-associated miR-512-3p modulates angiogenesis in pediatric Moyamoya disease by targeting ARHGEF3.

**DOI:** 10.1038/s41598-025-08796-4

**Published:** 2025-07-09

**Authors:** Eun Jung Koh, Seung Ah Choi, Youn Joo Moon, Heeyoung Lee, Ji Hoon Phi, Seung-Ki Kim

**Affiliations:** 1 Division of Pediatric Neurosurgery, Pediatric Clinical Neuroscience Center, Seoul National University Children’s Hospital, Seoul, Korea; 2 Department of Neurosurgery, Seoul National University Hospital, Seoul National University College of Medicine, Seoul, Korea; 3 Center of Hospital Medicine, Seoul National University Hospital, Seoul, Korea; 4 Division of Child Cancer, SNUH Kun-hee Lee Child Cancer & Rare Disease Project, Seoul, Korea; 5 Neuroscience Research Institute, Seoul National University College of Medicine, Seoul, Korea; 6 Division of Pediatric Neurosurgery, Seoul National University Children’s Hospital, 101 Daehak-ro, Jongno-gu, Seoul, 03080 Republic of Korea

**Keywords:** Moyamoya disease, Extracellular vesicles, Endothelial colony-forming cells, MiR-512-3p, ARHGEF3, Cell biology, Molecular biology, Biomarkers, Diseases

## Abstract

Moyamoya disease (MMD) is a chronic cerebrovascular disorder and a leading cause of pediatric stroke. Extracellular vesicles (EVs) carrying microRNAs (miRNAs) play a pivotal role in intercellular communication within cerebrovascular diseases. This study aimed to identify specific miRNAs within plasma-derived EVs from MMD patients and investigate their functional implications. Study subjects included healthy controls (*N* = 13) and MMD patients (*N* = 23). EVs were isolated from plasma samples and characterized by transmission electron microscopy, nanoparticle tracking analysis, ExoView, RT-qPCR, and immunoblotting. miRNA profiles were assessed through NanoString analysis. Functional effects of miR-512-3p inhibition were evaluated in MMD endothelial colony-forming cells (ECFCs) by analyzing guanosine triphosphatase (GTPase) activity, tubule formation, and cell viability. MMD-derived EVs exhibited an upregulation of miR-512-3p compared to controls. Bioinformatics analysis identified RHO guanine nucleotide exchange factor 3 (ARHGEF3) as a potential target of miR-512-3p. Inhibition of miR-512-3p in MMD ECFCs resulted in increased expression of ARHGEF3 and its downstream effector RHOA, leading to enhanced GTPase activity and improved tubule formation, indicative of restored angiogenic function. Elevated levels of miR-512-3p within plasma-derived EVs may serve as a novel biomarker for MMD diagnosis. The modulation of ARHGEF3 and subsequent RHOA signaling by targeting miR-512-3p contributes to the dysregulated angiogenesis in MMD.

**Supplementary Information:**

The online version contains supplementary material available at 10.1038/s41598-025-08796-4.

Division of Pediatric Neurosurgery, Seoul National University Children’s Hospital, 101 Daehak-ro, Jongno-gu, Seoul 03080, Republic of Korea.

Tel: 82-2-2072-3084, Fax: 82-2-744-8459, E-mail: nsthomas@snu.ac.kr.

## Introduction

Moyamoya disease (MMD) is a progressive cerebrovascular disorder characterized by the bilateral occlusion of the supraclinoid internal carotid artery (ICA) and its major branches^1^. In two-thirds of pediatric patients, progression leads to chronic ischemic injury and recurrent strokes, ultimately diminishing cognitive and physical capabilities, significantly impacting the quality of life^2^. Early diagnosis and treatment are therefore crucial.

MMD is diagnosed via conventional cerebral angiography as a confirmatory test. However, this procedure is invasive, and sedation is essential in children. Although magnetic resonance imaging/angiography (MRI/A) is a noninvasive screening test, it tends to exaggerate the stenosis and underestimate moyamoya vessels^3^. Furthermore, it is difficult to distinguish MMD from other cerebrovascular disorders because of its limited specificity^4^.

Liquid biopsy has emerged as a practical, noninvasive diagnostic tool for various diseases, including cancer and vascular disorders^5,6^. This technique detects disease-specific genomes, transcripts, and proteomes in body fluids, facilitating early diagnosis. In pediatric MMD patients, retrieving tissue from affected cerebral arteries is not feasible, making liquid biopsy a viable alternative for diagnosis. Plasma, being the most accessible body fluid, is particularly relevant. Circulating biomolecules could illuminate pathogenic cascades and uncover biomarkers for early diagnosis, risk stratification, and treatment monitoring^7^.

MicroRNAs (miRNAs), 22 nucleotide non-coding regulators of post-transcriptional state, are key candidates^8^. They reflect cellular identity, travel systemically in protein or vesicular carrier, and are dysregulated in diverse vasculopathies^9,10^. Among the most stable carriers are extracellular vesicles (EVs), nano- to micro-sized bilayer particles which are secreted by all cell type and found in nearly all biofluids^11^. EV membranes shield nucleic acids, lipids, and proteins from enzymatic degradation, conferring superior stability over free-circulating counterparts^12,13^. Mounting evidence shows that EV-encapsulated miRNAs (EV-miRNAs) outperform total plasma miRNAs as diagnostic biomarkers in vascular disorders^14^.

Despite the well-recognized vascular remodeling that defines MMD, comprehensive EV-miRNA profiling in pediatric patients and functional validation of candidate miRNAs remain conspicuously scarce. Few EV-based biomarkers have entered clinical evaluation for MMD, and the molecular mechanisms linking circulating signals to intracranial arteriopathy are largely speculative. To bridge these gaps, we profiled miRNAs in plasma-derived EVs from children with MMD and investigated how dysregulated miRNAs influence disease-relevant pathways.

## Methods

We presented a figure outlining the overall study design (Supplementary Figure S1).

### Human blood collection and plasma isolation

Peripheral blood samples were collected from healthy volunteers (control, *N* = 13) and MMD patients (*N* = 23; Table 1 and Supplementary Table S1). MMD diagnosis was confirmed via cerebral angiography, with clinical staging and RNF213 variant status verified by sequencing. Blood from MMD patients was collected from a radial artery catheter during indirect bypass surgery under general anesthesia. Due to ethical constraints regarding blood collection from children, blood samples from young adults with no history of stroke, high blood pressure, chronic disease, or smoking were used as control. Blood was collected into ethylenediaminetetraacetic acid (EDTA) tubes, with 10 mL of blood extracted for plasma isolation while the remaining 30 ml was used for mononuclear cell isolation. Samples were centrifuged at 2,500 rpm for 15 min. Plasma isolated from the blood was then transferred to a 15 mL conical tube and further centrifuged at 2,000 g for 20 min. This study was approved by the Seoul National University Hospital Institutional Review Board (IRB #1904-096-1027) and conducted in accordance with the Helsinki Declaration.

**Table 1:** Baseline characteristics of control subjects and Moyamoya disease patients.

Variable	Control (*N* = 13)		MMD (*N* = 23)	*P*-value
Age, yr — median (IQR)		23 (21–25)	7 (5–10)	< 0.0001
Sex — male, N (%)		8 (61.5)	12 (52.2)	0.73
Sex — female, N (%)		5 (38.5)	11 (47.8)	–
RNF213 p.R4810K genotype — N (%)				< 0.0001
• G/G (wild-type)		13 (100)	3 (13.0)	
• G/A (heterozygous)		0 (0)	18 (78.3)	
• A/A (homozygous)		0 (0)	2 (8.7)	

###  Cell cultures

Endothelial colony-forming cells (ECFCs) were isolated and cultured from the peripheral blood of both control individuals (*N* = 9) and MMD patients (*N* = 6) following previously established protocols^15,16^. Human umbilical vein endothelial cells (HUVECs) were purchased from ATCC (Manassas, VA). Both ECFCs and HUVECs were cultured in Endothelial Growth Medium-2 (EGM-2; Lonza, Walkersville, MD) and maintained at 37 °C in a humidified incubator with 5% CO₂. For experimental use, ECFCs at passage 6 were utilized. Prior to experiments, flow cytometry and fluorescence analysis were performed to characterize the cells.

### Isolation of EVs

EVs in plasma from control healthy volunteers (*N* = 10) and MMD patients (*N* = 14) were isolated using the total exosome isolation kit (Invitrogen, Carlsbad, CA) according to the manufacturer’s protocol. Briefly, frozen plasma samples were equilibrated to room temperature. After centrifugation of 1.2 mL plasma at 10,000 g for 20 min, residual cells and cell debris were removed. Supernatant (1 mL) was transferred into a new tube, and 0.5 volumes of 1 × PBS were added. To remove the bulk of protein from plasma, 0.05 volumes of protease K were added, and the tubes were incubated at 37 ℃ for 10 min. Then, 0.2 volumes of exosome precipitation reagent were added into the tube. The tube was thoroughly mixed and incubated at 4 ℃ for 30 min. This was followed by centrifugation at 10,000 g for 5 min, after which the supernatant was discarded and EVs were contained in a pellet.

Additionally, EVs were isolated from the conditioned media of MMD ECFCs using a miRCURY exosome isolation kit (Exiqon, Woburn, MA). ECFCs were cultured to 90% confluency in EGM-2 with exosome-free FBS (System Biosciences, Palo Alto, CA). Briefly, ECFCs were maintained for 3 days until 90% confluency in EGM-2 with exosome-free FBS (System Biosciences, CA, USA). Conditioned media (10 mL) was harvested and centrifuged at 3,200 × g for 15 min to remove residual cells and debris. The supernatant (8 mL) was mixed with precipitation buffer (5:2 ratio), incubated at 4 °C for 1 h, and centrifuged at 3,200 × g for 30 min to isolate EVs.

### Transmission electron microscopy (TEM)

EVs from control (*N* = 2) and MMD (*N* = 5) were used for TEM. TEM was employed to examine EV morphology and size, as previously described. Isolated EVs were mixed with 4% paraformaldehyde and embedded in a formvar-carbon-coated grid at room temperature. Then, the EVs were washed, fixed, stained, embedded, dried, and visualized with TEM (JEOL, Peabody, MA).

### Nanoparticle tracking analysis (NTA)

EVs from control (*N* = 3) and MMD (*N* = 5) were used for NTA. EV concentration and size distribution were assessed using the NanoSight NS300 (Malvern Instruments, Malvern, UK) as described previously^17^. EVs were diluted with autoclaved distilled water to obtain an optimum particle concentration of 20–150 per one frame, and EVs were diluted with 1 mL. The samples were added into a chamber held on a light microscope, and the images were captured in triplicate over a 60 s period at 10 frames/sec. The record provided the distribution of the number of particles per unit volume classified by size.

### ExoView analysis

The ExoView system (NanoView Biosciences, Brighton, MA) was used to analyze EVs, following the manufacturer’s protocol^9^. For the detection of tetraspanins, EVs from control (*N* = 5) and MMD (*N* = 8) were used. Diluted plasma samples (1:10 dilution) were incubated overnight on microarray chips (NanoView) coated with capture antibodies against CD81-CF555 (green, NanoView), CD63-CF647 (red, NanoView), CD9-CF488 (blue, NanoView), or a negative control IgG1 (NanoView).

For the detection of miRNAs, EVs from control (*N* = 5) and MMD (*N* = 6) were used. A molecular beacon approach was employed. Diluted plasma samples (1:5 dilution) or conditioned media (1:2 dilution) were incubated overnight on microarray chips (NanoView) coated with a capture molecular beacon for [FAM]miR-512-3p[BHQ1] (Bioneers, Dajeon, Korea) or a negative control (Bioneers). EVs were captured with antibodies targeting CD9 via the miR-512-3p molecular beacon and subsequently imaged. The number of positive particles was quantified using an ExoView R100 reader with nScan 2.8.4 acquisition software (NanoView), and the data were analyzed using NanoViewer 3.0.

### Protein extraction and quantification

 Proteins were extracted from EVs and cells using distinct methods. EV proteins were isolated using the Exo2D kit (EXOSOMEplus, Gyeonggi-do, Republic of Korea) according to the manufacturer’s instructions, while cellular proteins were extracted using RIPA buffer (GenDEPOT, Barker, TX, USA) to ensure efficient lysis and solubilization. Protein concentrations were determined using the BCA Protein Assay Kit (ThermoFisher Scientific, Rockford, IL), and the extracted proteins were used for western blot analysis and Exo-Check antibody array.

### EV marker profiling

To assess the presence of exosomal markers, the Exo-Check exosome antibody array (SBI, Palo Alto, CA) was utilized. This array includes eight exosomal markers (CD63, CD81, ALIX, FLOT1, ICAM1, EpCAM, ANXA5, and TSG101) along with four control spots (two horseradish peroxidase (HRP)-positive controls, one blank spot, and GM130 as a cis-Golgi contamination marker). EVs from control (*N* = 1) and MMD (*N* = 1) were used. A total of 50 µg of EV proteins was labeled according to the manufacturer’s protocol, and excess labeling reagent was removed using the purification columns provided in the kit. The labeled EV lysates were applied to the array membrane and incubated overnight at 4 °C. Following a series of washes and incubation with the detection buffer, signals were visualized using a chemiluminescent substrate, and images were acquired using the Amersham Imager 680 (GE Healthcare Bio-Sciences AB, Uppsala, Sweden).

### Western blot analysis

Western blotting was performed as described previously^16^ to evaluate the expression of proteins in EV and cellular lysates. EVs from control (*N* = 8) and MMD (*N* = 8) were used. The membranes were incubated with primary antibodies at the following dilutions: flotillin-1 (1:1,000; Abcam, Cambridge, MA), cytochrome C (1:1,000; Cell Signaling Technology, Danvers, MA), ARHGEF3 (1:1,000; Abcam), GXYLT1 (1:1,000; ThermoFisher), SPOP (1:1,000; Abcam), SMNDC1 (1:1,000; Abcam), and β-actin (1:2,000; Abcam). Flotillin-1 was used as an exosomal marker, while cytochrome C served as a negative control for EVs. β-Actin was used as a loading control for normalization in cellular lysates. Following incubation with HRP-conjugated secondary antibodies, membranes were developed using the Novex™ ECL Chemiluminescent Substrate Reagent Kit (Life Technologies, Carlsbad, CA). Protein bands were detected using either X-ray film or the Chemi Imager 680 system (GE, Amersham Marlborough, MA). Densitometric quantification was performed using ImageJ software, with protein band intensities normalized to β-Actin for cellular lysates, and values were calculated relative to the respective negative control (NC)-inhibitor or NC-mimic controls.

### RNA isolation

Total RNAs, including miRNAs, were isolated from EVs and ECFCs using the miRNeasy Kit (Qiagen, Hilden, Germany) as per established protocols^9^. RNA quality and quantity were assessed with a Nanodrop 2000 Spectrophotometer and Agilent 2100 Bioanalyzer (Agilent Technologies, Santa Clara, CA).

### NanoString miRNA analysis

NanoString nCounter platform (NanoString Technologies, Inc., Seattle, WA) was used to analyze EV-miRNAs using 800 human v3 miRNA expression assays. EVs from control (*N* = 10) and MMD (*N* = 14) were analyzed. For each sample, 100 ng of total RNA isolated from plasma-derived EVs was used as input, and 3 µL was loaded per reaction. Differential expression analysis was performed to identify miRNAs that were either upregulated or downregulated between control and MMD samples. miRNAs with |log2 fold change| ≥ 1 and *p* < 0.05 were considered significantly differentially expressed miRNAs (DEmiRs) and selected for further analysis. Principal component analysis (PCA) was used to visualize the global distribution of DEmiRs, and a volcano plot was used for annotation of significantly altered miRNAs. Receiver operating characteristic (ROC) analysis was used to evaluate the discriminatory power of DEmiRs.

### Integrative analysis of miRNAs and mRNAs

An integrative analysis was performed, combining miRNA NanoString data with mRNA array data^9^. DEmiRs were analyzed to identify potential targets by integrating gene expression data from control (*N* = 4) and MMD (*N* = 7) ECFCs^16^ with predictions from TargetScan (v.8.0, targetscan.org)^18,19^. Predicted miRNA-mRNA interactions were visualized using the Functional Enrichment Analysis Tool (FunRich, funrich.org)^20^. Enrichment analysis was conducted to assess the presence of mRNAs in biological pathways curated in databases such as the Kyoto Encyclopedia of Genes and Genomes (KEGG, genome.jp/kegg)^21^ and WikiPathways (wikipathways.org)^22^. P values from enrichment analysis were adjusted using the false discovery rate method.

### Real-time quantitative polymerase chain reaction (RT–qPCR)

RT-qPCR was employed to validate the expression levels of miRNAs and mRNAs. ECFCs from control (*N* = 4) and MMD (*N* = 4) were used. miRNA quantification (miR-512-3p, miR-320e) was performed using miScript SYBR Green qPCR kits (Qiagen), with RUN6B as the reference gene. For mRNA validation, including ARHGEF3, TOX, PCDH10, PFKP, GXYLT1, MBNL1, ARL5B, SPOP, SMNDC1, PTBP2, RHOA, RHOB, CDC42, RAC1, RAC2, and RASIP1, TaqMan probes (Applied Biosystems, Waltham, MA) were utilized according to the manufacturer’s instructions, with GAPDH as the reference gene. Relative expression levels were calculated using the 2^−ΔΔCt method, with normalization to RNU6B for miRNAs and GAPDH for mRNAs. All reactions were run in triplicate, and results are presented as mean ± standard deviation. All experiments were conducted in triplicate.

### miRNA transfection

miRNA inhibitors, mimics, and their corresponding NCs were obtained from Qiagen. Cells were seeded in 6-well plates at a density of 2 × 10⁵ cells/well and incubated for 16 h prior to transfection. Transfection was performed using lipofectamine RNAiMAX (Invitrogen) according to the manufacturer’s instructions. For miR-512-3p inhibition, MMD ECFCs were transfected with miR-512-3p inhibitor (*N* = 6) at a final concentration of 50–100 nM, or with the corresponding NC-inhibitor (*N* = 5) at the same concentration, depending on cell condition and transfection efficiency. For miR-512-3p overexpression, HUVECs were transfected with 50 nM miR-512-3p mimic or the corresponding NC-mimic. Transfection efficiency was evaluated 24 h post-transfection by RT-qPCR. All concentrations were determined based on preliminary dose–response optimization. All experiments were performed in at least three independent replicates.

### Guanosine triphosphatase (GTPase) activity assay

GTPase activity was measured using a colorimetric GTPase assay kit (Sigma–Aldrich) following the manufacturer’s instructions. MMD ECFCs with NC-inhibitor (*N* = 3) and MMD ECFCs with miR-512-3p inhibitor (*N* = 4) were used. After transfection, the cells were harvested and sonicated in cold assay buffer. Reaction mixtures contained 20 µL of Assay Buffer and 10 µL of 4 mM guanosine triphosphate (GTP). The mixtures were incubated with samples for 30 min at room temperature to terminate. The optical density (OD) was measured using an ELISA reader at 620 nm. The GTPase activity (mU/L) was calculated by the following formula: [Pi] (mM) × 40 µL ÷ [10 µL × reaction time (min)]. This value was corrected by dividing by the protein quantitative value (mg/L). The unit of GTPase activity was expressed as mU/mg in the graph.

### Cell viability analysis

Cell viability was assessed using the EZ-Cytox cell viability assay kit (Daeil Biotech, Suwon, Korea). MMD ECFCs with NC-inhibitor (*N* = 3) and MMD ECFCs with miR-512-3p inhibitor (*N* = 4) were used. Cells were seeded at a density of 1 × 10^4^/well in 96-well plates and incubated for 24, 48, and 72 h. After incubation, the cells were treated with EZ-Cytox solution, and OD was measured at 450 nm using an ELISA reader. Experiments were repeated in triplicate.

### Tubule formation analysis

Tubule formation was performed using Matrigel (BD Biosciences, San Jose, CA). MMD ECFCs with NC-inhibitor (*N* = 3) and MMD ECFCs with miR-512-3p inhibitor (*N* = 4) were used. Matrigel was added to 48-well plates and incubated at 37 °C. Cells were seeded at a density of 2 × 10^4^/well on Matrigel-coated 48-well plates and incubated for 18 h at 37 °C and 5% CO_2_. Capillary structures were observed and photographed under an inverted phase-contrast microscope (Zeiss, Oberkochen, Germany). Each experiment was repeated at least three times.

### Statistical analysis

Statistical comparisons between two groups were made using Student’s unpaired T-test or Mann-Whitney U test for continuous variables. For multiple comparisons, one-way or two-way ANOVA followed by Bonferroni correction was used. Chi-Squared test or Fisher’s exact test was used for categorical variables. For PCA, normalized and standardized expression values were used to derive the first two principal components, which were then plotted to examine sample-level variance. ROC curves and the area under the ROC curve (AUC) were calculated to assess diagnostic accuracy. The differences were considered significant when P values were less than 0.05.

## Results

### Characterization of EVs in control and MMD plasma samples

**Fig. 1:**
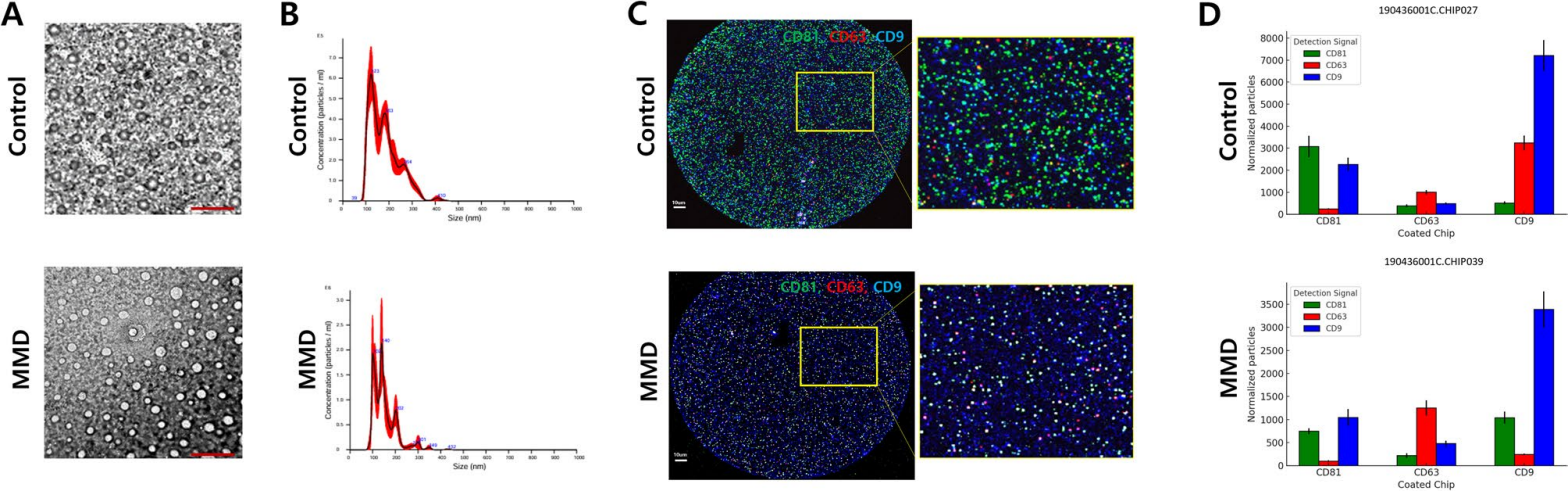
Characterization of extracellular vesicles (EVs) isolated from control and moyamoya disease (MMD) plasma. (**A**) Representative transmission electron microscopy (TEM) images showing the typical morphology of exosomes isolated from control (*N* = 2) and MMD (*N* = 5) plasma. Scale bars = 2 μm. (**B**) Nanoparticle tracking analysis (NTA) of EVs from control (*N* = 3) and MMD (*N* = 5) plasma, depicting particle concentration and size distribution. (**C**) ExoView analysis illustrating the enrichment of EV surface markers (CD81, CD63, and CD9) in control (*N* = 5) and MMD (*N* = 8) plasma EVs. Coexpression of these markers was assessed using fluorescently labeled antibodies: CD81 (green), CD63 (red), and CD9 (blue). The yellow square portion is a magnified image. Scale bars = 10 μm. (**D**) Bar graphs present mean ± SD values, analyzed using Student’s t-test.

We confirmed that EVs isolated from both control healthy volunteers and MMD plasma samples met the identification criteria outlined by the Minimal Information for Studies of Extracellular Vesicles (MISEV)^23^. TEM imaging revealed the typical morphology of EVs from both groups, showing round-shaped membrane vesicles with diameters ranging from 50 to 200 nm (Fig. 1A). NTA revealed that the total number of EV particles was significantly higher in control individuals compared to MMD patients (control vs. MMD: 1.048 × 10^9^ ± 2.401 × 10^8^ vs. 5.653 × 10^8^ ± 1.989 × 10^8^, *p* = 0.011), although no significant difference in particle size was observed (control vs. MMD: 135.6 ± 21.92 nm vs. 144.1 ± 5.07 nm, *p* = 0.238; Fig. 1B).

EV protein markers were characterized using three distinct methods. First, the ExoView affinity microarray system identified EV tetraspanin markers (CD81, CD63, and CD9) in both control and MMD-derived EVs (Fig. 1C). Both groups exhibited the highest particle counts on the CD9-coated chip with CD9 detection (7,208 ± 700 in control EVs; 3,391 ± 389 in MMD EVs), indicating CD9 as the predominant surface marker. Most tetraspanin signals were lower in MMD EVs compared to controls across multiple capture/detection combinations. However, CD63 detection on the CD63-coated chip was increased in MMD EVs (1,253 ± 166) relative to control EVs (1,005 ± 96), suggesting a relative enrichment of CD63-positive vesicles (Fig. 1D and Supplementary Table S2). We conducted pairwise comparison of detection markers stratified by study group and coated chip to assess the significance of statistical difference between two markers (Supplementary Table S3). Second, we confirmed that the positive EV marker flotillin-1 was present in most EV samples, whereas the negative control cytochrome C was absent in both control and MMD plasma-derived EVs (Supplementary Fig. S2A). We performed quantitative analysis of flotillin-1 expression. The expression of flotillin-1 was greater in EVs from plasma of control individuals than in those from plasma of MMD patients (*p* = 0.0089; Supplementary Fig. S2B). Finally, to evaluate a broader range of EV markers, we utilized the ExoCheck antibody array, which allows for simultaneous detection of multiple markers. All exosome markers and positive controls exhibited signals in both control and MMD EVs. The blank spot showed no signal, and the negative control (GM130) produced only a negligible signal, confirming the presence of exosome with minimal contamination (Supplementary Fig. S2C). No significant differences were observed between the two groups.

#### Identification of DEmiRs in EVs from MMD plasma

**Fig. 2:**
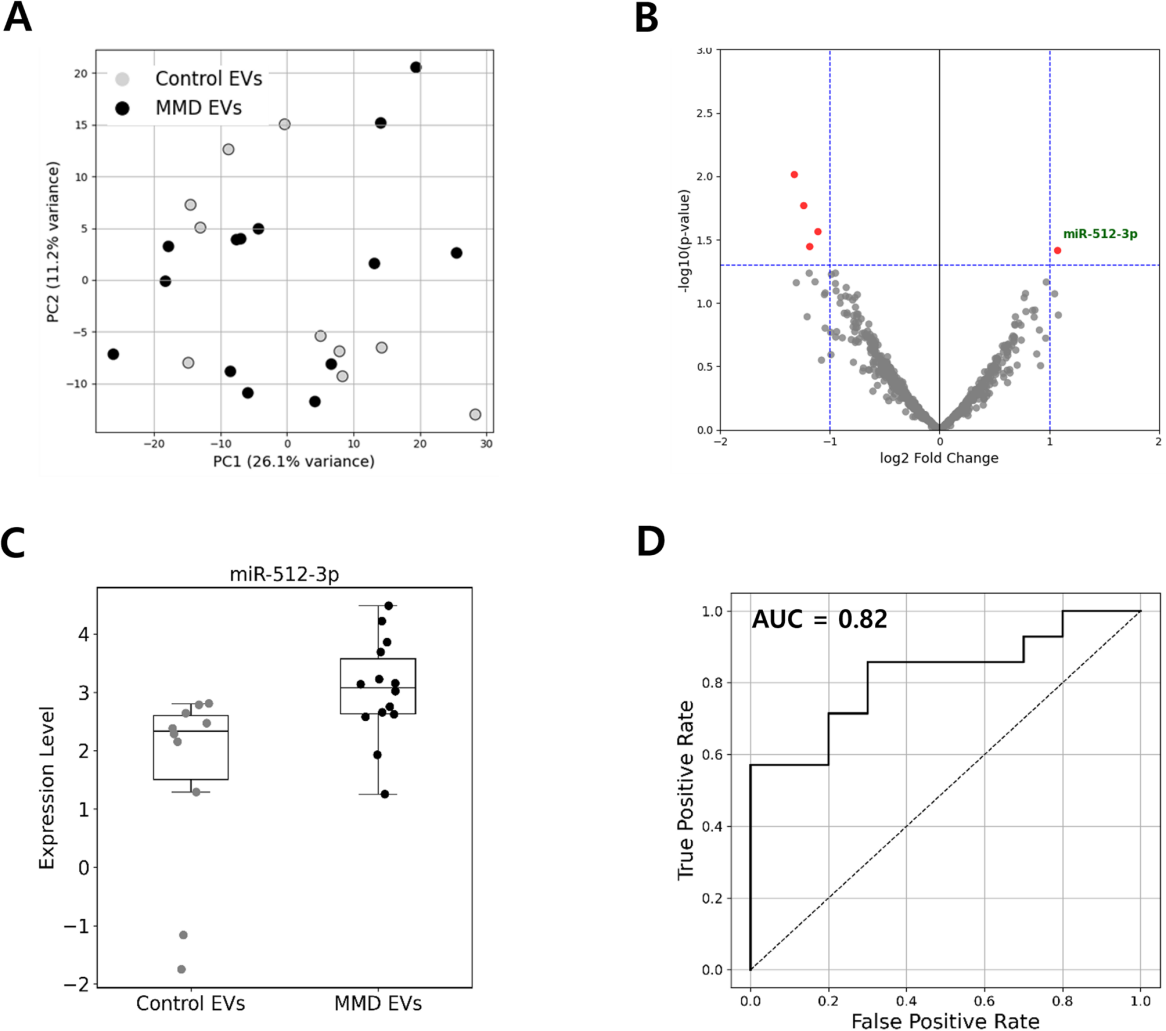
Identification of diagnostic miR-512-3p in extracellular vesicles (EVs) isolated from moyamoya disease (MMD) plasma. (**A**) Principal component analysis (PCA) demonstrating partial separation between control EVs and MMD EVs based on global miRNA expressions. (**B**) Volcano plot highlighting differentially expressed miRNAs between groups (|log2FC| ≥ 1, *p* < 0.05), with hsa-miR-512-3p marked in red. (**C**) Scatter plot of hsa-miR-512-3p expression levels across control (*N* = 10) and MMD (*N* = 14) samples. (**D**) Receiver operating characteristic (ROC) curve of hsa-miR-512-3p based on NanoString data, demonstrating its diagnostic potential with an AUC of 0.82.

**Fig. 3:**
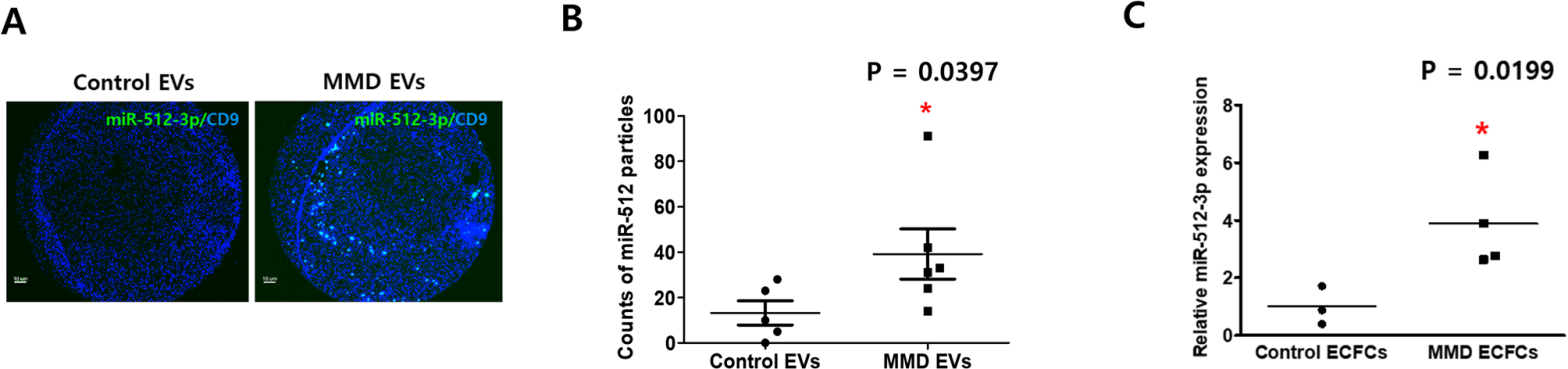
Validation of miR-512-3p in extracellular vesicles (EVs) and endothelial colony-forming cells (ECFCs) from moyamoya disease (MMD) patients. (A) NanoView analysis showing colocalization of miR-512-3p (green) with CD9 (blue) in EVs (control *N* = 5, MMD *N* = 8). Scale bars = 10 μm. (B) Quantification of colocalized particle counts (control *N* = 5, MMD *N* = 6), presented as mean ± SD. Statistical significance was determined using Student’s t-test. *P < 0.05. (C) Relative expression of miR-512-3p in MMD ECFCs (*N* = 4) compared to control ECFCs (*N* = 4), presented as mean ± SD values from RT-qPCR analysis. *P < 0.05.

To visualize expression differences between controls and MMD patients, we conducted a PCA (Fig. 2A) and generated a volcano plot (Fig. 2B) based on plasma EVs miRNA. PCA revealed partial separation between control EVs and MMD EVs, while hierarchical clustering (Supplementary Fig. S3A, Supplementary Table S4) showed incomplete group segregation, likely reflecting biological heterogeneity and the limited sample size. The volcano plot identified multiple differentially expressed miRNAs, among which hsa-miR-512-3p was the most significantly upregulated in MMD EVs. In contrast, miR-219a-3p, miR-3136-5p, miR-1268a, and miR-320e were downregulated (Table 2). Given the advantages of upregulated miRNAs in detection and targeting, miR-512-3p was prioritized as a biomarker candidate. Moreover, scatter plot (Fig. 2C) and ROC curve analysis (Fig. 2D) based on the NanoString dataset further demonstrated the discriminatory potential of miR-512-3p, yielding an AUC of 0.82. We also presented scatter plot (Supplementary Fig. S3B), ROC curve analysis (Supplementary Fig. S3C) of each four downregulated miRNAs, and multivariable ROC curve combining miR-512-3p, miR-320e, and miR-1268a (Supplementary Fig. S3D). Validation using a molecular beacon-based ExoView assay confirmed that miR-512-3p levels were approximately fourfold higher in MMD EVs compared to control EVs (*p* = 0.0397; Fig. 3A and B). A similar increase was observed in MMD ECFCs (*p* = 0.0199; Fig. 3C), supporting consistency across EVs and ECFCs.

**Table 2:** Profiling of differentially expressed MiRNAs in extracellular vesicles from plasma of patients with Moyamoya disease.

miRNA	Expression	logFC	AveExpr	*t*	*p*-value
hsa-miR-512-3p	up	1.07659	2.451814	2.13819	0.038527
hsa-miR-219a-3p	down	−1.10808	3.092496	−2.28829	0.027362
hsa-miR-3136-5p	down	−1.23795	3.519189	−2.48697	0.017058
hsa-miR-1268a	down	−1.32360	3.826419	−2.71337	0.009704
hsa-miR-320e	down	−3.71402	8.325576	−4.43591	6.77E-05

logFC, Log_2_ fold change; AveExpr, The average log_2_ expression level.

### Identification of the target genes of miR-512-3p in EVs from MMD plasma

Building on prior mRNA array data from MMD ECFCs^16^ (Supplementary Fig. S4A, Supplementary Table S5), we investigated the predicted target genes of the five key EV-miRNAs, focusing on the functional role of miR-512-3p in MMD ECFCs (Supplementary Fig. S4B). The primary molecular functions identified were categorized as molecular function unknown, RNA binding, catalytic activity, and ubiquitin-specific protease activity. Fourteen downregulated genes with binding sites for miR-512-3p were identified (Table 3). Among these predicted targets, the top 10 were validated using RT-qPCR.

**Table 3:** The target genes of miR-512-3p in endothelial colony-forming cells from patients with Moyamoya disease.

Target symbol	logFC	*p*-value	Key to Abbreviations and Symbols	Mechanism
ARHGEF3	−0.94857	6.44E-05	RHO guanine nucleotide exchange factor 3	GTPases regulation
TOX	−0.9426	5.64E-07	Thymocyte selection-associated high mobility group box	DNA binding, regulating T-cell development
PCDH10	−0.87165	8.89E-08	Protocadherin-10	Cell adhesion
PFKP	−0.54767	0.027887	Phosphofructokinase	Catalyzation of the phosphorylation of D-fructose 6-phosphate to fructose 1,6-bisphosphate by ATP
GXYLT1	−0.38038	0.00239	Glucoside xylosyltransferase 1	UDP-xylosyltransferase activity
MBNL1	−0.35169	0.005029	Muscleblind like splicing regulator 1	Pre-mRNA alternative splicing regulation
ARL5B	−0.28548	0.004888	ADP ribosylation factor like GTPase 5B	GTPases regulation
SPOP	−0.25718	0.049932	Speckle-type BTB/POZ protein	Ubiquitin protein ligase binding
SMNDC1	−0.24844	0.006545	Survival motor neuron domain containing 1	Spliceosome assembly
PTBP2	−0.23215	0.030718	Polypyrimidine tract binding protein 2	Negative regulation of exons splicing.
SFMBT1	−0.17826	0.000555	Scm-like with four MBT domains 1	Histone-binding protein
CUL1	−0.14358	0.028045	Cullin 1	Cell cycle
RSBN1L	−0.12393	0.0146	Round spermatid basic protein 1 like	Demethylation of methylated lysine residues
MBNL2	−0.11524	0.035313	Muscleblind like splicing regulator 2	Pre-mRNA alternative splicing regulation

logFC, Log_2_ fold change.

**Fig. 4:**
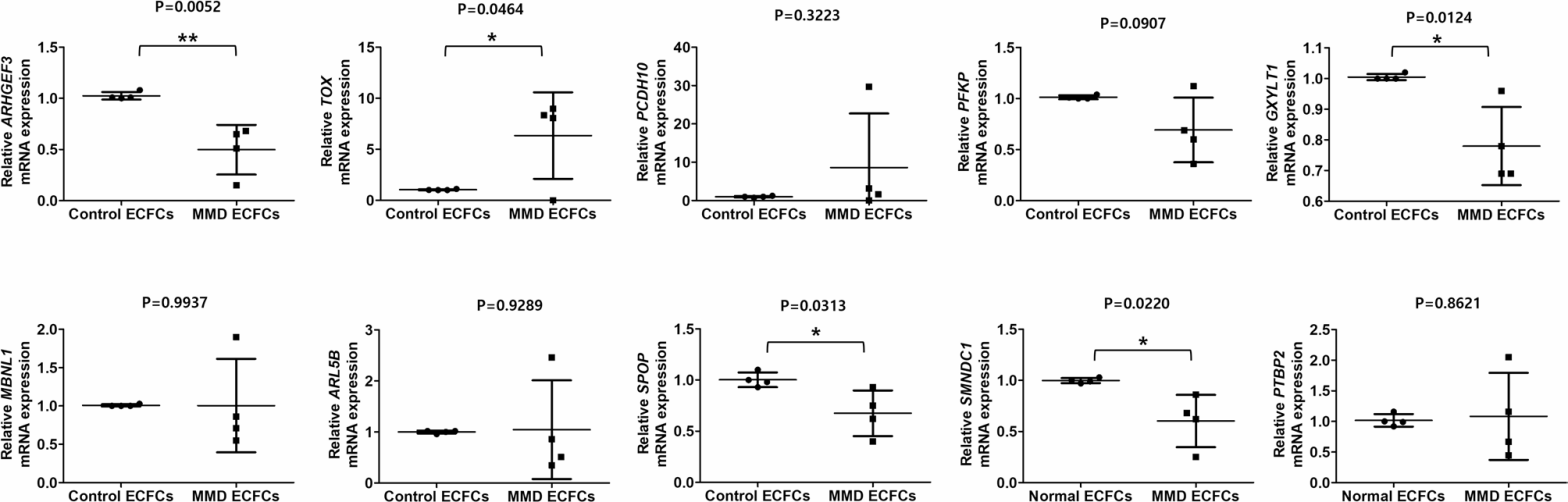
Verification of the target genes of miR-512-3p in endothelial colony-forming cells (ECFCs) from patients with moyamoya disease (MMD). Fourteen genes predicted as miR-512-3p targets were identified through integrative analysis, with the expression patterns of 10 genes validated by RT-qPCR. ARHGEF3, GXYLT1, SPOP, and SMNDC1 exhibited significantly lower expression in MMD ECFCs (*N* = 4) compared to control ECFCs (*N* = 4). The data is presented as mean ± SD and analyzed using Student’s t-test. *P < 0.05, **P < 0.01.

We validated the expression of these ten miR-512-3p target genes in control and MMD ECFCs by performing RT-qPCR. RHO guanine nucleotide exchange factor 1 (ARHGEF3), glucoside xylosyltransferase 1 (GXYLT1), speckle-type BTB/POZ protein (SPOP), and survival motor neuron-related splicing factor 30 (SMNDC1) were significantly lower expressed in MMD ECFCs compared to control ECFCs, consistent with the experimental data (Fig. 4). Interestingly, thymocyte selection-associated high mobility group box (TOX) was upregulated in MMD ECFCs, contrary to the predictions, while the other genes showed no significant differences. ARHGEF3, identified as the most significant gene in RT-qPCR validation, is known to regulate metabolic processes through a GTPase mechanism^24–26^. We verified interaction between miR-512-3p and the 3′ untranslated region of ARHGEF3 using TargetScan database (Supplementary Fig. S5).

#### Regulation of target genes by miR-512-3p Inhibition in MMD ECFCs

**Fig. 5:**
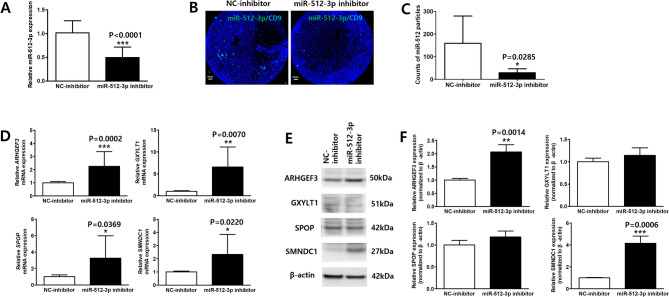
Upregulation of reduced target genes by miR-512-3p inhibition in endothelial colony-forming cells (ECFCs) from patients with moyamoya disease (MMD). (MMD ECFCs with NC-inhibitor *N* = 5, MMD ECFCs with miR-512-3p inhibitor *N* = 6) (A) RT-qPCR analysis showing significantly reduced miR-512-3p levels in MMD ECFCs transfected with a miR-512-3p inhibitor compared to miRNA inhibitor negative control (NC-inhibitor). (B and C) ExoView analysis demonstrating a significant reduction in miR-512-3p particles in EVs from MMD ECFCs following miR-512-3p inhibition. Scale bars = 10 μm. (D) RT-qPCR results indicating a significant increase in ARHGEF3, GXYLT1, SPOP, and SMNDC1 expression in MMD ECFCs after miR-512-3p inhibition. (E and F) Western blot analysis showing significant increase in ARHGEF3 and SMNDC1 protein expression, with no significant changes of GXYLT1 and SPOP protein levels. The data is presented as mean ± SD and analyzed using Student’s t-test. *P < 0.05, **P < 0.01, ***P < 0.001.

The biological impact of miR-512-3p inhibition was explored in MMD ECFCs. Transfection with a miR-512-3p inhibitor for 48 h significantly reduced miR-512-3p expression by over 50%, indicating effective inhibition (*p* < 0.0001; Fig. 5A). This led to an approximate five-fold decrease in miR-512-3p particles in EVs secreted by MMD ECFCs (*p* = 0.0285; Fig. 5B and C). To explore downstream effects, we assessed whether miR-512-3p inhibition could alter the expression of four selected target genes in MMD ECFCs. miR-512-3p inhibition resulted in the upregulation of all four target genes, with ARHGEF3 showing the most pronounced increase (*p* = 0.0002; Fig. 5D). Western blot analysis demonstrated a significant increase in ARHGEF3 (*p* = 0.0014) and SMNDC1 (*p* = 0.0006) expression following miR-512-3p inhibition, while no significant changes were observed in GXYLT1 and SPOP protein levels (Fig. 5E and F).

#### Functional validation of miR-512-3p

**Fig. 6:**
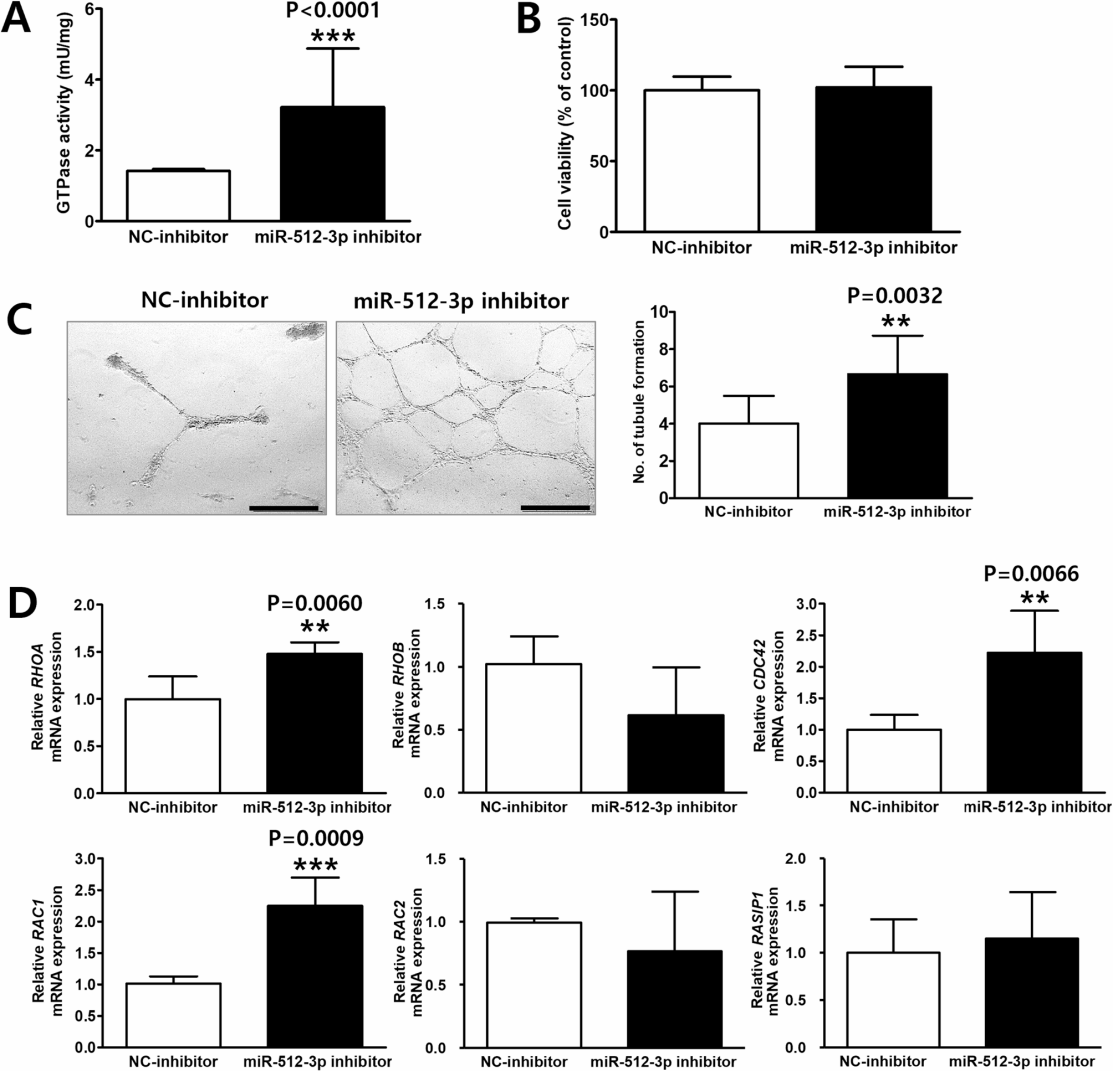
Biological impacts of miR-512-3p depletion on endothelial colony-forming cells (ECFCs) from patients with moyamoya disease (MMD). (MMD ECFCs with NC-inhibitor *N* = 3, MMD ECFCs with miR-512-3p inhibitor *N* = 4) (A) GTPase assay showing significantly increased GTPase activity following miR-512-3p inhibition in MMD ECFCs. (B) Cell viability assay demonstrating unaffected viability of MMD ECFCs by miR-512-3p inhibition. (C) Tubule formation assay showing improved angiogenic function in MMD ECFCs after miR-512-3p inhibition. Scale bars = 500 μm. The graph represents the quantitative analysis of tubule branch numbers. (D) RT-qPCR analysis demonstrating that miR-512-3p inhibition upregulated small RHO GTPases, including RHOA, CDC42, and RAC1. The data is presented as mean ± SD and analyzed using Student’s t-test. **P < 0.01, ***P < 0.001.

ARHGEF3 plays a crucial role in various cellular processes through its GTPase activity^27–29^. Consistent with this, GTPase activity increased significantly by 2.3-fold in MMD ECFCs following miR-512-3p inhibition (NC-inhibitor vs. miR-512-3p inhibitor: 1.4 ± 0.1 vs. 3.2 ± 1.7 mU/mg, *p* < 0.0001; Fig. 6A). miR-512-3p inhibition did not impact the viability of MMD ECFCs (*p* > 0.05; Fig. 6B) but significantly increased tubule formation by 1.7-fold (NC-inhibitor vs. miR-512-3p inhibitor: 4.0 ± 1.5 vs. 6.7 ± 2.1 tubules, *p* = 0.0032; Fig. 6C). Furthermore, analysis of small RHO GTPases (RHOA, RHOB, CDC42, RAC1, RAC2, and RASIP1), which are linked to angiogenesis^30–34^revealed that RHOA (*p* = 0.0060), CDC42 (*p* = 0.0066), and RAC1 (*p* = 0.0009) were upregulated after miR-512-3p inhibition, while RHOB, RAC2, and RASIP1 showed no significant changes (Fig. 6D). To further delineate the regulatory role of miR-512-3p, we employed a gain-of-function approach by transfecting HUVECs with a miR-512-3p mimic or a corresponding NC-mimic. RT-qPCR analysis confirmed a significant increase in miR-512-3p expression following transfection (*p* < 0.0001; Supplementary Fig. S6A). Consistent with miR-512-3p-mediated post-transcriptional suppression, Western blot analysis revealed a significant reduction in ARHGEF3 protein levels (*p* = 0.0001; Supplementary Fig. S6B and S6C). Functionally, miR-512-3p overexpression led to a modest but significant increase in cell viability (*p* < 0.0001; Supplementary Fig. S6D) and a marked impairment of tubule formation capacity (NC-mimic vs. miR-512-3p mimic: 8.4 ± 1.7 vs. 3.5 ± 3.0 tubules, *p* = 0.004; Supplementary Fig. S6E and S6F), indicating a suppressive role of miR-512-3p in angiogenesis. These results support the conclusion that miR-512-3p modulates endothelial cell function, as its inhibition enhances tubule formation, whereas its overexpression leads to the opposite effects.

## Discussion

Identifying a universal, disease-specific, miRNA biomarker remains challenging due to cohort differences in ethnicity, age, gender and clinical settings^35,36^. Furthermore, the lack of reproducibility in miRNA biomarker studies across different research groups may stem from pre-analytical variability in sample processing, platform-specific technical biases, and inconsistent normalization strategies^37–39^. Both free-circulating miRNAs and EV-miRNAs have been investigated as diagnostic markers of MMD in liquid biopsy samples. Several studies have extracted EV-miRNAs from plasma or CSF and compared patients and control groups, revealing differentially expressed miRNAs in the patient cohort (Supplementary Table S6)^7,40–44^. Pinpointing common key miRNAs throughout these investigations was challenging, the pathways enriched in their target genes mainly centered on vascular remodeling. Unlike most studies screening EV-miRNAs in MMD which reported broad expression panels without converging on a single mechanistic driver^7,40,42,43^, we move beyond descriptive profiling and provide a testable mechanism that may explain the defective angiogenesis of MMD. Our study compared EV-miRNAs from MMD plasma with those from control plasma and identified five differentially expressed miRNAs. We focused on upregulated miRNAs for biomarker identification, given their detection and targeting advantages. Among these, miR-512-3p was the only significantly upregulated miRNA in EVs from MMD plasma. To elucidate its role, we conducted in vitro experiments and identified ARHGEF3 as a key target gene of miR-512-3p.

ARHGEF3, a member of the RHO guanine nucleotide exchange factor (RHOGEF) family, enhances RHO GTPase activity by facilitating the conversion of GTP to GDP. It is broadly expressed in blood cells and the brain^27^, and selectively activates RHOA and RHOB but not RHOC or other small GTPases^28,29,45,46^. RHO proteins and their effectors are key mediators of vascular endothelial growth factor (VEGF)-induced angiogenesis^25^. Our findings confirm the upregulation of RHOA following miR-512 inhibition in MMD ECFCs, implicating ARHGEF3 in angiogenesis and RHOA/RHO-associated coiled-coil kinase (ROCK) signaling^47,48^. The RHOA/ROCK pathway enhances vascular permeability, promotes matrix metalloproteinase (MMP)-mediated extracellular matrix (ECM) degradation via mitogen-activated protein kinase (MAPK) signaling, and facilitates endothelial migration, proliferation, and morphogenesis by promoting the G1/S cell cycle transition^24,25,32,49,50^.

Pathogenic variants of Diaphanous related formin 1 (DIAPH1) were identified in non-East-Asian MMD patients. DIAPH1 encodes mDia1, whose GTPase-biding domain associates with active RHOA to drive actin-filament polymerization. DIAPH1 variants weaken the RHOA-mDia1 interaction and disturb cytoskeletal remodeling^51^. Guanylate cyclase soluble subunit alpha-3 (GUCY1A3) has also emerged as a potent causative gene in MMD^52^. GUCY1A3 encodes alpha1-subunit of soluble guanylate cyclase (sGC). sGC, activated by nitric oxide (NO), produces cyclic guanosine monophosphate (cGMP) that maintains the relaxation of vascular smooth muscle cell (VSMC) by inhibiting RHOA/ROCK signaling^53^. Recent works on MMD have shown that GUCY1A3 mutation dampen NO-sGC-cGMP signaling, promoting VSMC proliferation and intimal hyperplasia^54^. The dysfunction of mDia1, insufficiency of sGC activity and ARHGEF3 downregulation by miR-512-3p in MMD converge mechanistically, all shifting the balance away from RHOA activation via distinct molecular levers.

Enhanced versus impaired tubule formation in MMD models highlights the tension between compensatory angiogenesis and maladaptive vascular remodeling. RNF213 knock-down promotes exuberant endothelial cell sprouting through Hippo-YAP/TAZ–VEGFR2 signaling, echoing the fragile collaterals seen^55^. In contrast, iPS-derived ECs from patients with the p.4810 K mutation showed blunted tubulogenesis and cytoskeletal disruption owing to integrin-β3 loss, paralleling vessel destabilization and intimal hyperplasia^56^. The pathogenic duality in MMD angiogenesis plausibly reflects stage-dependent shifts: early hypoxia stimulates VEGF-driven EC proliferation, whereas chronic inflammation and MMP–mediated ECM degradation later undermines vessel integrity. These findings necessitate therapeutic strategies targeting temporal-specific mechanisms. RHOA/ROCK signaling can contribute to both the early vascular proliferation phase and the later vessel disintegration. What we captured in this study is that ARHGEF3 likely contributes to angiogenesis through the activation of RHOA/ROCK signaling, and its downregulation by miR-512-3p may be involved in the development of aberrant angiogenesis in MMD.

CDC42 and RAC1, angiogenesis-related RHO GTPases that are not direct ARHGEF3 targets, were likewise up-regulated, suggesting indirect modulation by miR-512-3p or the inter-RHO crosstalk documented among RHOGEFs and GAPs^46,57^. RHOB, an ARHGEF3-responsive GTPase, showed a nonsignificant decrease relative to RHOA after miR-512-3p inhibition. Because RHOB dampens RHOA activity during VEGF-driven endothelial sprouting^57^, this suggests a complex interplay between RHOA and RHOB in vascular remodeling, with potential antagonistic interactions.

Increasing evidence indicates that RNF213 expression is co-regulated by many circulating miRNAs, and that the dysregulation of this network directly contributes to the impaired angiogenesis in MMD. An early serum miRNome study identified 16 miRNAs that cooperatively suppress RNF213, establishing the inaugural mechanistic link between miRNA imbalance and MMD pathology^58^. Subsequent work revealed that let-7c binds to RNF213 3’-UTR and represses its expression, positioning let-7c as a potential diagnostic marker^59^. A proteo-transcriptomic analysis demonstrated that the plasma miRNA signature is reshaped by the presence of RNF213 p.R4810K variant^7^, highlighting the need for genotype-stratified investigations; this genotype-dependent pattern was independently reproduced in monozygotic twins^60^. RNF213 is not listed as a direct target of miR-512-3p in the miRNA database, and our limited sample size prevented us from comparing expression across different RNF213 genotypes; future multivariate miRNA panels and functional studies should incorporate RNF213 genotype to fully elucidate the molecular crosstalk driving MMD pathogenesis^7,58–60^.

Our study has several limitations. First, the control group was not age-matched to the pediatric MMD patients, which raises concerns about the influence of aging on EV populations and miRNA profiles. Ethical and practical constraints make it difficult to recruit healthy pediatric controls. Second, there was little overlap between the samples with EV and ECFC measurements. Because most EV and ECFC samples were not matched pairs from the same individuals, direct correlation analysis between miRNA and target gene expression could not be performed. Fold change values for miR-512-3p and ARHGEF3 between plasma-derived EVs and ECFCs were discrepant. These results may reflect tissue-specific regulation of miRNA-mRNA interactions, or differences in biological complexity and detection sensitivity between cell-free and cell-based systems. Furthermore, the relatively small sample size limits the statistical power of our findings, underscoring the need for larger, independent pediatric cohort to further validate miR-512-3p as a diagnostic marker. Lastly, we utilized ECFCs derived from pediatric MMD patients as an in vitro cell model, which inherently has limitations due to their low abundance in peripheral blood. It is certainly possible that ECFCs serve as a source of miR-512-3p; however, because they comprise only a small fraction of peripheral blood cells, their contribution to the overall elevation of miR-512-3p expression is likely minimal. ECFCs represent a feasible alternative given the scarcity of suitable animal models for MMD and the difficulties associated with obtaining vascular tissue samples from pediatric patients. Numerous studies have emphasized the critical roles of ECFCs in angiogenesis and vascular remodeling^61,62^. In addition, their significance as a valuable in vitro cell model in MMD research has been highlighted in numerous studies, demonstrated by reduced cell numbers, impaired tubule formation, and increased senescence or apoptosis in ECFCs from pediatric MMD patients compared to controls^15,16,63,64^.

This study explored plasma EV-miRNAs from pediatric MMD patients and confirmed their functional roles in MMD ECFCs. Our findings indicate that miR-512-3p within plasma EVs presents potential as a diagnostic biomarker for MMD. The suppression of ARHGEF3, a target gene of miR-512-3p, is implicated in the dysregulated angiogenesis observed in MMD by modulating RHOA signaling pathways. Conversely, inhibition of miR-512-3p, which upregulates ARHGEF3, may facilitate the restoration of normal angiogenesis.

## Electronic supplementary material

Below is the link to the electronic supplementary material.

Supplementary Material 1

Supplementary Material 2

Supplementary Material 3

Supplementary Material 4

Supplementary Material 5

Supplementary Material 6

Supplementary Material 7

Supplementary Material 8

Supplementary Material 9

Supplementary Material 10

Supplementary Material 11

## Data Availability

The data that supports the findings of this study is available from the corresponding author upon reasonable request.

## Abbreviations

ARHGEF3: RHO guanine nucleotide exchange factor 3
CDC42: Cell division control protein 42 homolog
cGMP: cyclic guanosine monophosphate
DEmiRs: Differentially expressed miRNAs
DIAPH1: Diaphanous related formin 1
ECFCs: Endothelial colony-forming cells
ECM: Extracellular matrix
EVs: Extracellular vesicles
EV-miRNA: Extracellular vesicle encapsulated miRNA
GTPase: Guanosine triphosphatase
GUCY1A3: Guanylate cyclase 1 soluble subunit alpha-3
GXYLT1: Glucoside xylosyltransferase 1
ICA: Internal carotid artery
MAPK: Mitogen-activated protein kinase
MISEV: Minimal Information for Studies of Extracellular Vesicles
MMD: Moyamoya disease
MMP: Matrix metalloproteinase
NC: Negative control
NTA: Nanoparticle tracking analysis
OD: Optical density
PCA: Principal component analysis
RAC1: Ras-related C3 botulinum toxin substrate 1
RHOA: Ras homolog family member A
ROCK: Rho-associated coiled-coil kinase
SMNDC1: Survival motor neuron domain containing 1
SPOP: Speckle-type BTB/POZ protein
TEM: Transmission electron microscopy
VEGF: Vascular endothelial growth factor
VSMC: Vascular smooth muscle cell
