# Supplementary material for: Plasma extracellular vesicle-associated miR-512-3p modulates angiogenesis in pediatric Moyamoya disease by targeting ARHGEF3
Source: Sci Rep. 2025 Jul 9;15:24655. doi: 10.1038/s41598-025-08796-4 (PMC12241339; doi:10.1038/s41598-025-08796-4)
Supplement: Supplementary file 3 — Supplementary Material 3 [file 41598_2025_8796_MOESM3_ESM.docx]

Supplementary Table S1. Clinical characteristics and experimental usage of blood from control healthy volunteers and patients with Moyamoya disease

| Sample ID | Sex | Age (yr) | Symptoms | MRI finding | Suzuki grade (R/L) | RNF213 p.R4810K genotype | TEM | NTA | WB | ExoView | ECFCs | EV miR-512-3p expression |
| --- | --- | --- | --- | --- | --- | --- | --- | --- | --- | --- | --- | --- |
| C1 | m | 21 |  |  |  | G/G |  |  |  | ○ |  | 2.642688000 |
| C2 | f | 21 |  |  |  | G/G |  |  |  |  | ○ | -1.749982519 |
| C3 | m | 21 |  |  |  | G/G |  |  | ○ | ○ |  | 2.382594519 |
| C4 | m | 22 |  |  |  | G/G |  |  | ○ |  |  | 1.161997092 |
| C5 | m | 25 |  |  |  | G/G |  |  | ○ | ○ | ○ |  |
| C6 | m | 21 |  |  |  | G/G |  |  |  |  |  | 2.153851518 |
| C7 | f | 24 |  |  |  | G/G | ○ | ○ |  |  | ○ | 2.787177040 |
| C8 | f | 24 |  |  |  | G/G |  | ○ | ○ |  |  | 1.291281646 |
| C9 | m | 19 |  |  |  | G/G |  | ○ | ○ |  |  | 2.471821599 |
| C10 | m | 25 |  |  |  | G/G |  |  |  |  |  | 2.287411667 |
| C11 | f | 23 |  |  |  | G/G |  |  | ○ |  |  |  |
| C12 | m | 25 |  |  |  | G/G |  |  | ○ | ○ |  |  |
| C13 | f | 25 |  |  |  | G/G | ○ |  | ○ | ○ | ○ | 2.809203346 |
| M1 | m | 9 | TIA | No infarct | 2/2 | G/A |  |  | ○ | ○ |  | 1.928819215 |
| M2 | f | 6 | TIA | No infarct | 2/2 | G/A |  |  | ○ | ○ | ○ | 3.156084519 |
| M3 | f | 11 | TIA | No infarct | 1/2 | G/A |  |  | ○ | ○ |  | 3.021535146 |
| M4 | f | 10 | TIA | No infarct | 3/3 | G/G |  |  |  | ○ |  | 2.580167651 |
| M5 | m | 11 | TIA | No infarct | 4/4 | G/A |  |  |  |  |  | 3.693157131 |
| M6 | f | 11 | Involuntary movement | No infarct | 3/4 | G/A |  |  |  | ○ |  | 3.138955658 |
| M7 | f | 5 | Hemiparesis | Infarct | 4/4 | G/A |  |  |  |  |  | 2.658588127 |
| M8 | f | 3 | Hemiparesis | Infarct | 3/3 | G/G |  |  |  |  |  | 2.623743366 |
| M9 | f | 7 | TIA | No infarct | 3/2 | G/A |  |  |  | ○ |  | 3.861731660 |
| M10 | f | 1 | Seizure | Infarct | NA | G/G |  |  |  |  |  | 4.222672333 |
| M11 | f | 7 | TIA | No infarct | 4/4 | G/A |  |  |  | ○ |  | 4.486165913 |
| M12 | m | 5 | TIA | No infarct | 3/2 | G/A |  | ○ | ○ | ○ | ○ | 0.753754039 |
| M13 | m | 9 | TIA | No infarct | 4/4 | G/A | ○ |  |  | ○ |  | 1.253827642 |
| M14 | m | 9 | Hemiparesis | Infarct | 3/3 | G/A | ○ |  |  |  | ○ | 3.226297432 |
| M15 | m | 5 | TIA | No infarct | 2/4 | A/A |  |  | ○ |  |  |  |
| M16 | f | 4 | TIA | No infarct | 3/3 | G/A |  |  | ○ |  |  |  |
| M17 | m | 2 | Hemiparesis | Infarct | 4/4 | A/A | ○ | ○ | ○ | ○ |  |  |
| M18 | m | 8 | TIA | No infarct | 4/4 | G/A | ○ | ○ | ○ | ○ |  |  |
| M19 | m | 6 | TIA | No infarct | 4.4 | G/A |  |  |  |  | ○ |  |
| M20 | m | 10 | TIA | No infarct | 3/3 | G/A |  |  |  |  | ○ |  |
| M21 | m | 16 | Hemiparesis | Infarct | 3/2 | G/A |  |  |  |  | ○ |  |
| M22 | m | 7 | TIA | No infarct | 3/2 | G/A |  |  |  |  | ○ |  |
| M23 | f | 16 | Hemiparesis | Infarct | 4/4 | G/A |  |  |  | ○ | ○ |  |

C, Control healthy volunteer; M, Moyamoya disease; m, Male; f, Female; TIA, Transient ischemic attack; MRI, Magnetic resonance imaging; R, Right hemisphere; L, Left hemisphere; NA, Not available; RNF213, Ring Finger Protein 213 gene; TEM, Transmission electron microscopy; NTA, Nanoparticle tracking analysis; WB, Western blot; ECFCs, Endothelial colony-forming cells; EV, Extracellular vesicle**Supplementary Table S2. Summary of ExoView tetraspanin capture and detection profiles in plasma-derived extracellular vesicles**

| Coated chip | Detection signal | Control EVs | MMD EVs | P value |
| --- | --- | --- | --- | --- |
| CD81 | CD81 | 3,078 ± 481 | 746 ± 64 | P < 0.0001 |
| CD81 | CD63 | 243 ± 22 | 96 ± 19 | P < 0.0001 |
| CD81 | CD9 | 2,262 ± 308 | 1,047 ± 182 | P < 0.0001 |
| CD63 | CD81 | 385 ± 64 | 217 ± 52 | P < 0.0001 |
| CD63 | CD63 | 1,005 ± 96 | 1,253 ± 166 | 0.00195 |
| CD63 | CD9 | 484 ± 48 | 479 ± 64 | 0.854 |
| CD9 | CD81 | 515 ± 69 | 1043 ± 131 | P < 0.0001 |
| CD9 | CD63 | 3,239 ± 336 | 244 ± 18 | P < 0.0001 |
| CD9 | CD9 | 7,208 ± 700 | 3,391 ± 389 | P < 0.0001 |

* Coated chip = Capture marker

**Supplementary Table S3. Pairwise comparison between tetraspanin markers**

| Group | Coated chip | Comparison | P value |
| --- | --- | --- | --- |
| Control EVs | CD81 | CD81 vs CD63 | P < 0.0001 |
| Control EVs | CD81 | CD81 vs CD9 | 0.000800578 |
| Control EVs | CD81 | CD63 vs CD9 | P < 0.0001 |
| Control EVs | CD63 | CD81 vs CD63 | P < 0.0001 |
| Control EVs | CD63 | CD81 vs CD9 | 0.002120162 |
| Control EVs | CD63 | CD63 vs CD9 | P < 0.0001 |
| Control EVs | CD9 | CD81 vs CD63 | P < 0.0001 |
| Control EVs | CD9 | CD81 vs CD9 | P < 0.0001 |
| Control EVs | CD9 | CD63 vs CD9 | P < 0.0001 |
| MMD EVs | CD81 | CD81 vs CD63 | P < 0.0001 |
| MMD EVs | CD81 | CD81 vs CD9 | 0.0008788350 |
| MMD EVs | CD81 | CD63 vs CD9 | P < 0.0001 |
| MMD EVs | CD63 | CD81 vs CD63 | P < 0.0001 |
| MMD EVs | CD63 | CD81 vs CD9 | P < 0.0001 |
| MMD EVs | CD63 | CD63 vs CD9 | P < 0.0001 |
| MMD EVs | CD9 | CD81 vs CD63 | P < 0.0001 |
| MMD EVs | CD9 | CD81 vs CD9 | P < 0.0001 |
| MMD EVs | CD9 | CD63 vs CD9 | P < 0.0001 |

**Supplementary Table S6. Studies investigating extracellular vesicle miRNAs in Moyamoya disease**

| Authors (Year) | Cohort | Specimen | Analysis | Key miRNA | Enriched pathway |
| --- | --- | --- | --- | --- | --- |
| Lee et al.  (2019) ^7^ | 12 MMD vs 12 controls (family members of MMD patients) | Plasma EVs | NGS  qRT-PCR | MMD-affected miRs; ↓  miR-100-5p  miR-122-5p  miR-1307-3p  miR-331-3p  miR-365a-3p  miR-370-3p  miR-9-5p | Immune response  Cell growth and differentiation  Signal transduction  Nervous system |
| Wang et al.  (2020) ^36^ | 31 MMD vs 31 controls | CSF exosomes | Microarray  qRT‑PCR | 4‑miR panel; ↑  miR‑3679‑5p  miR‑6165  miR‑6760‑5p  miR‑574‑5p | Cell‑adhesion  Cell‑junction organisation |
| Ota et al.  (2023) ^42^ | 8 MMD vs 4 ischemic controls | CSF EVs | NGS  qRT‑PCR | 153↑/98↓ miRs;  top:  miR‑421  miR‑361‑5p  miR‑320a  miR‑29b‑3p | Cytoplasmic stress‑granule assembly  RNA‑metabolism |
| Huang et al.  (2023) ^43^ | 9 MMD vs 10 non‑MMD | Plasma exosomes | NGS  qRT‑PCR | 1,002 DEmiRs;  10‑marker panel (e.g., miR‑1306‑5p, miR‑196b‑5p) | Axon guidance  Actin‑cytoskeleton regulation  MAPK signalling |
| Wang et al.  (2024) ^44^ | 10 MMD vs 10 controls | Peripheral‑blood exosomes | NGS  qRT‑PCR | 2‑miRs combo↑  miR‑328‑3p +  miR‑200c‑3p | Intercellular‑junction integrity  Impaired cytoskeletal remodeling  Fibroblast proliferation |
| Liu et al.  (2024) ^45^ | 6 MMD vs 3 controls | Plasma exosomes | in‑vitro  EC assays | miR‑125b‑5p  miR‑151a‑3p | Endothelial‑to‑mesenchymal transition  Migratory phenotype |
| Current study | 23 MMD vs 13 healthy controls | Plasma EVs | NanoString  qRT-PCR  in-vitro  ECFC assays | ↑miR-512-3p  ↓miR-320e  ↓miR-1268a  ↓miR-3136-5p  ↓miR-219a-2-3p | Common enriched pathway: molecular function unknown  miR-512-3p target gene ARHGEF3  →RhoA/ROCK signaling |
