# Supplementary figures and images for: Plasma extracellular vesicle-associated miR-512-3p modulates angiogenesis in pediatric Moyamoya disease by targeting ARHGEF3
Source: Sci Rep. 2025 Jul 9;15:24655. doi: 10.1038/s41598-025-08796-4 (PMC12241339; doi:10.1038/s41598-025-08796-4)

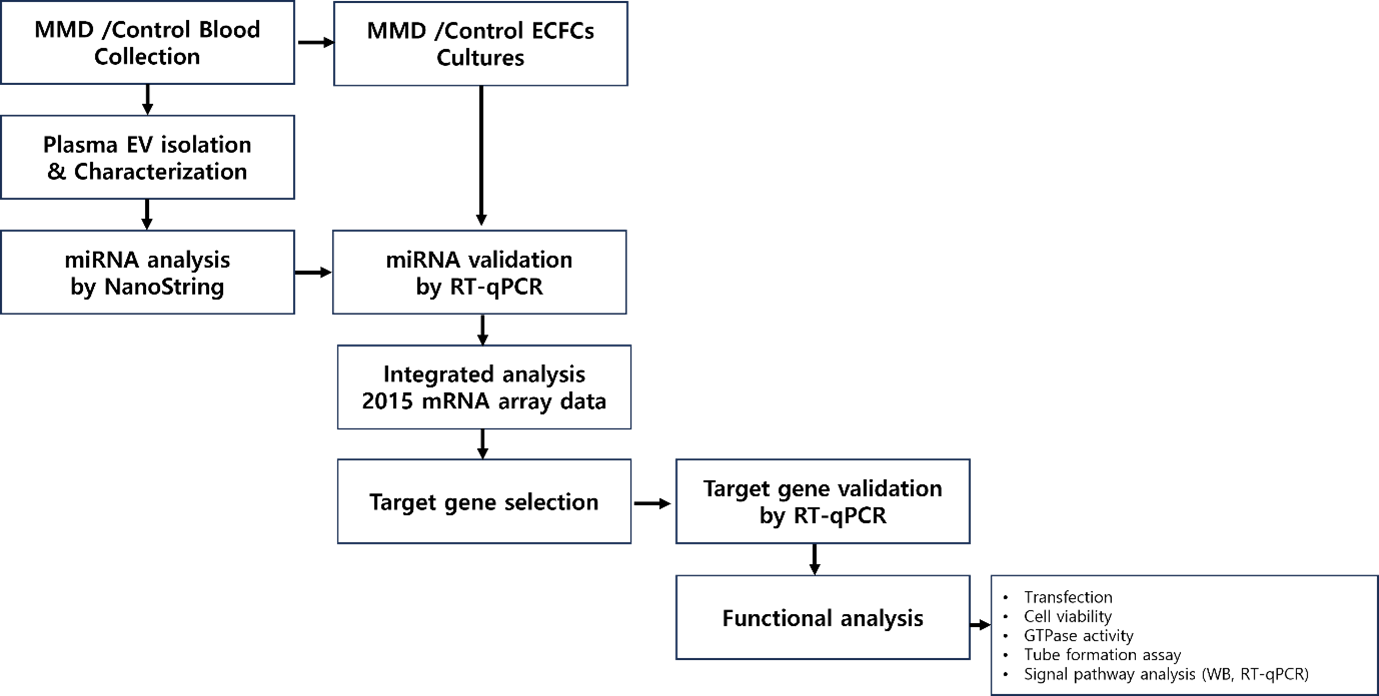

Supplement: Supplementary file 4 — Supplementary Material 4 [file 41598_2025_8796_MOESM4_ESM.tif]

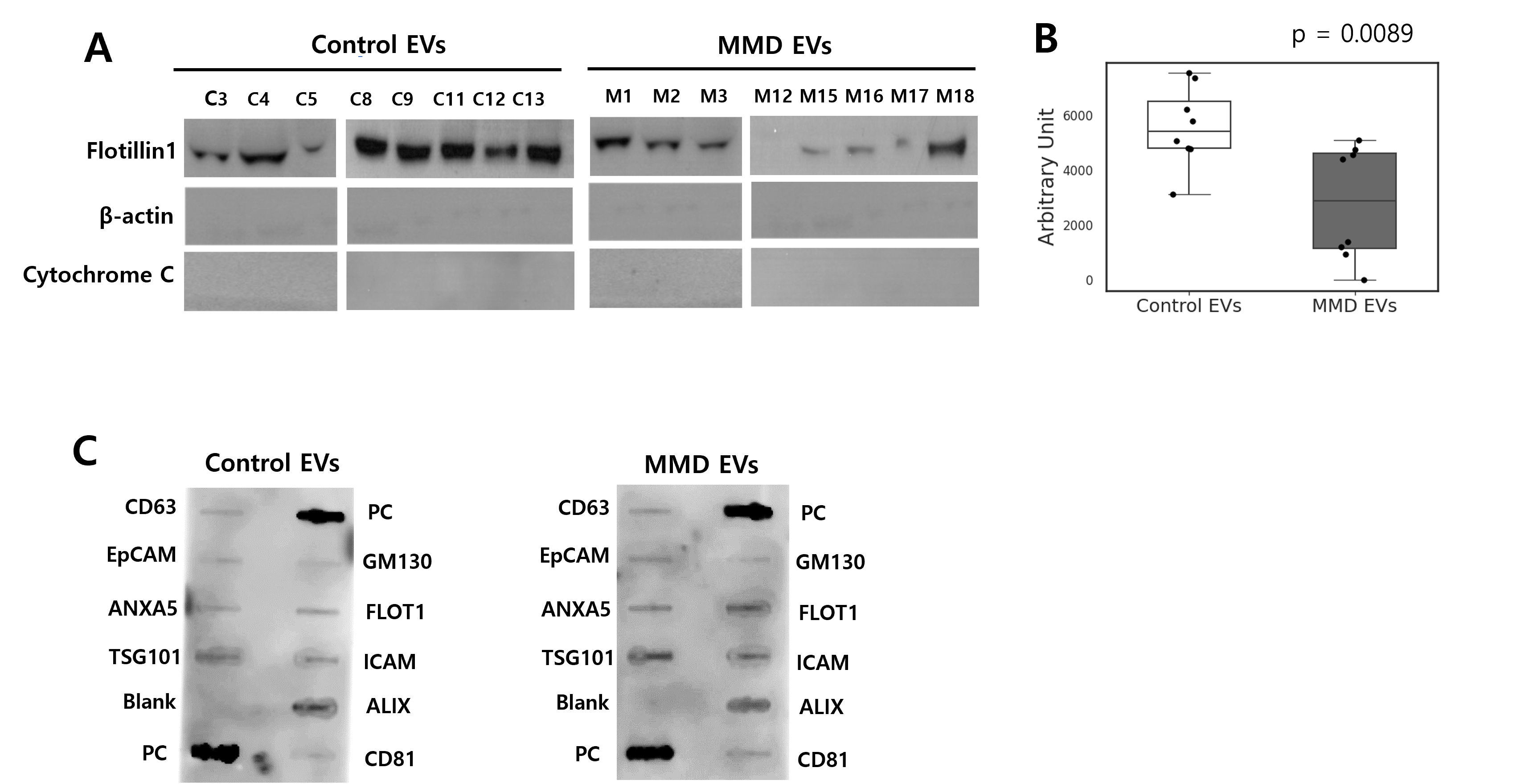

Supplement: Supplementary file 5 — Supplementary Material 5 [file 41598_2025_8796_MOESM5_ESM.tif]

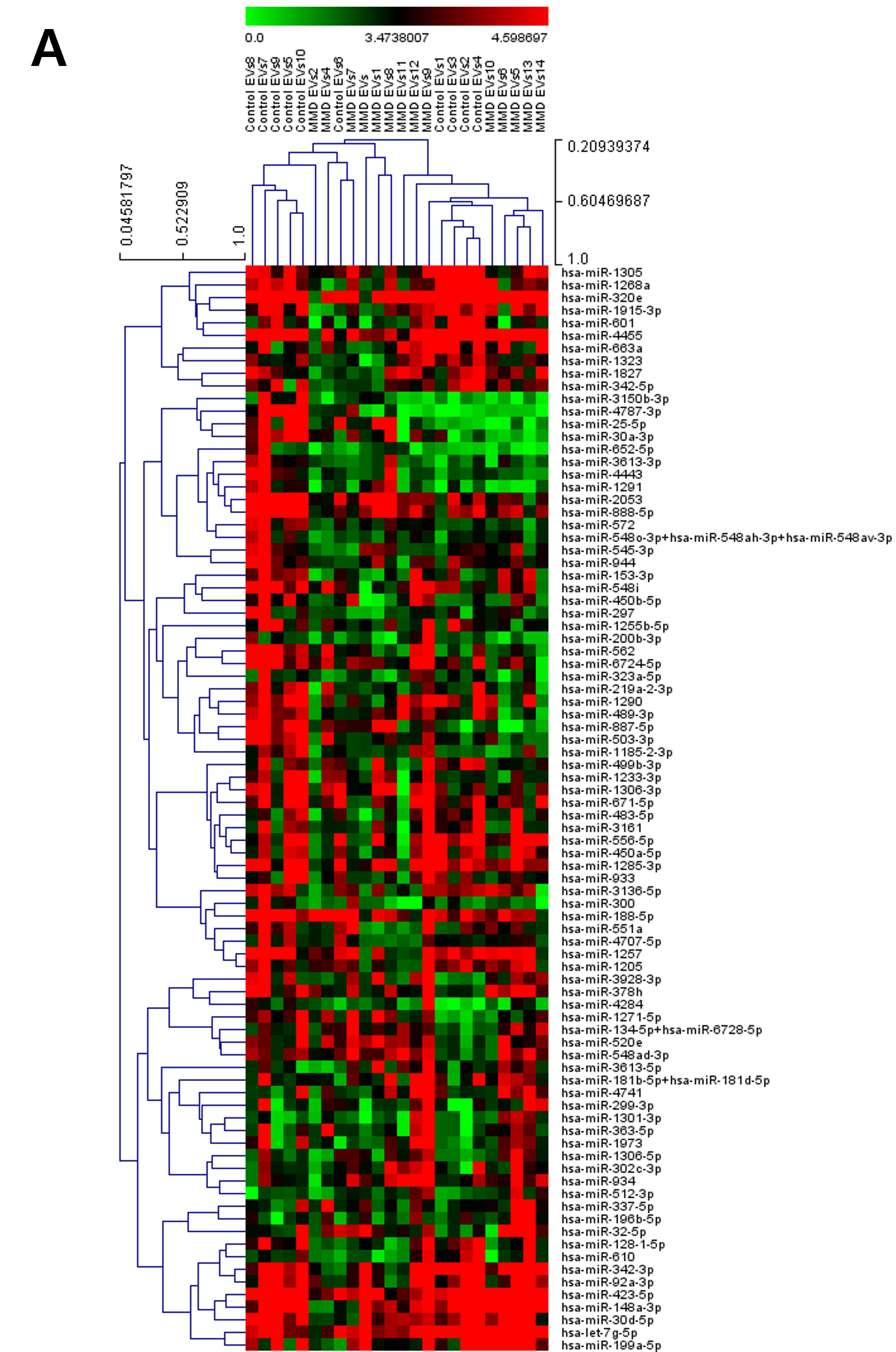

Supplement: Supplementary file 6 — Supplementary Material 6 [file 41598_2025_8796_MOESM6_ESM.tif]

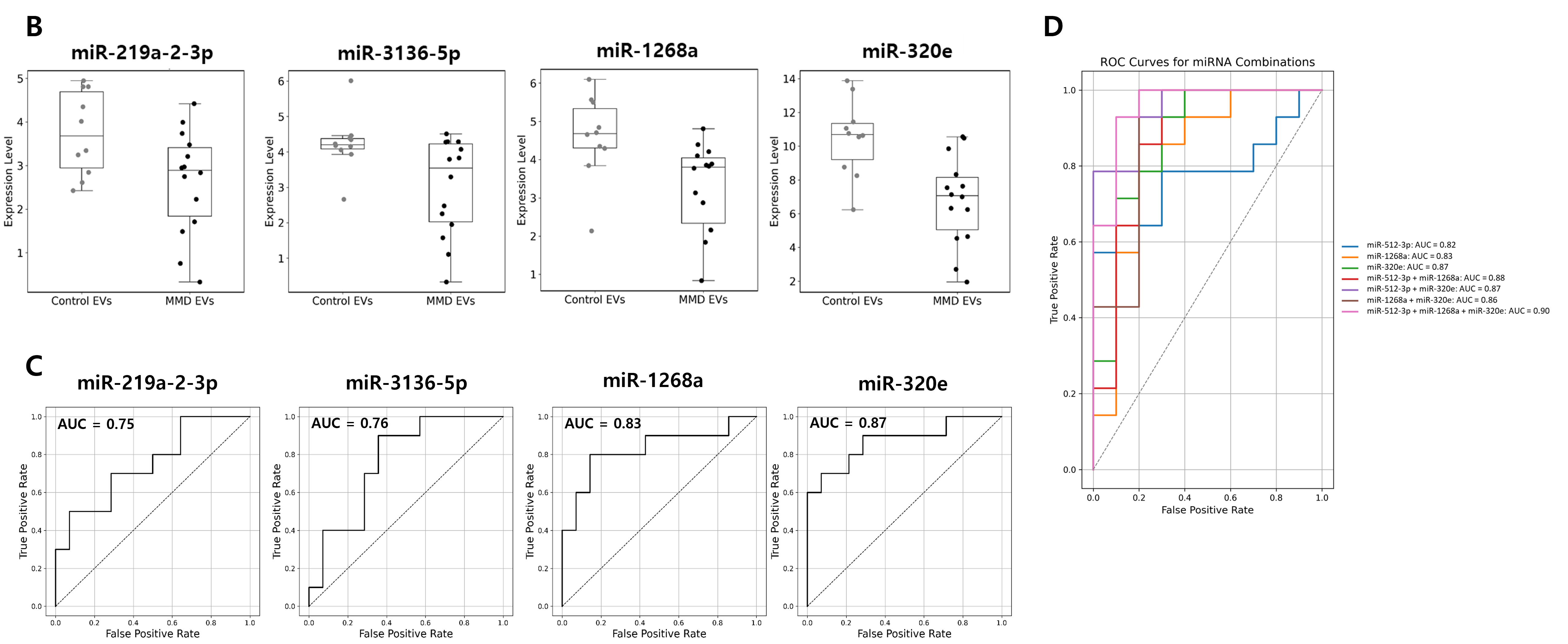

Supplement: Supplementary file 7 — Supplementary Material 7 [file 41598_2025_8796_MOESM7_ESM.tif]

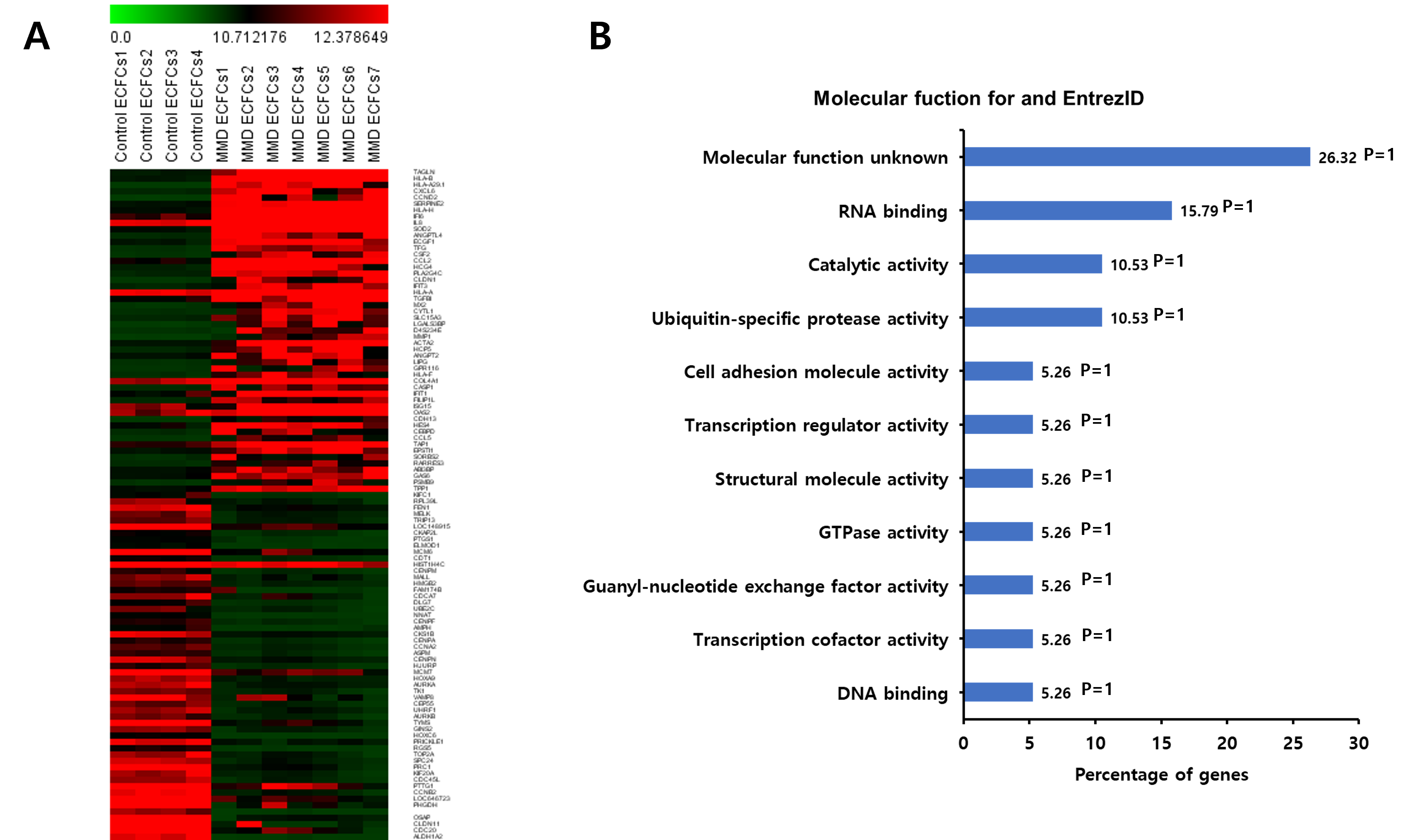

Supplement: Supplementary file 8 — Supplementary Material 8 [file 41598_2025_8796_MOESM8_ESM.tif]

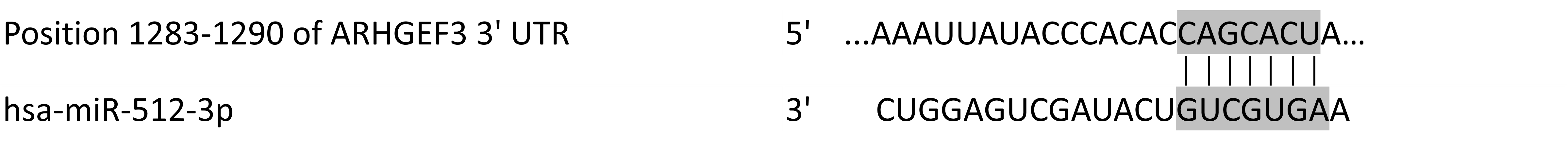

Supplement: Supplementary file 9 — Supplementary Material 9 [file 41598_2025_8796_MOESM9_ESM.tif]

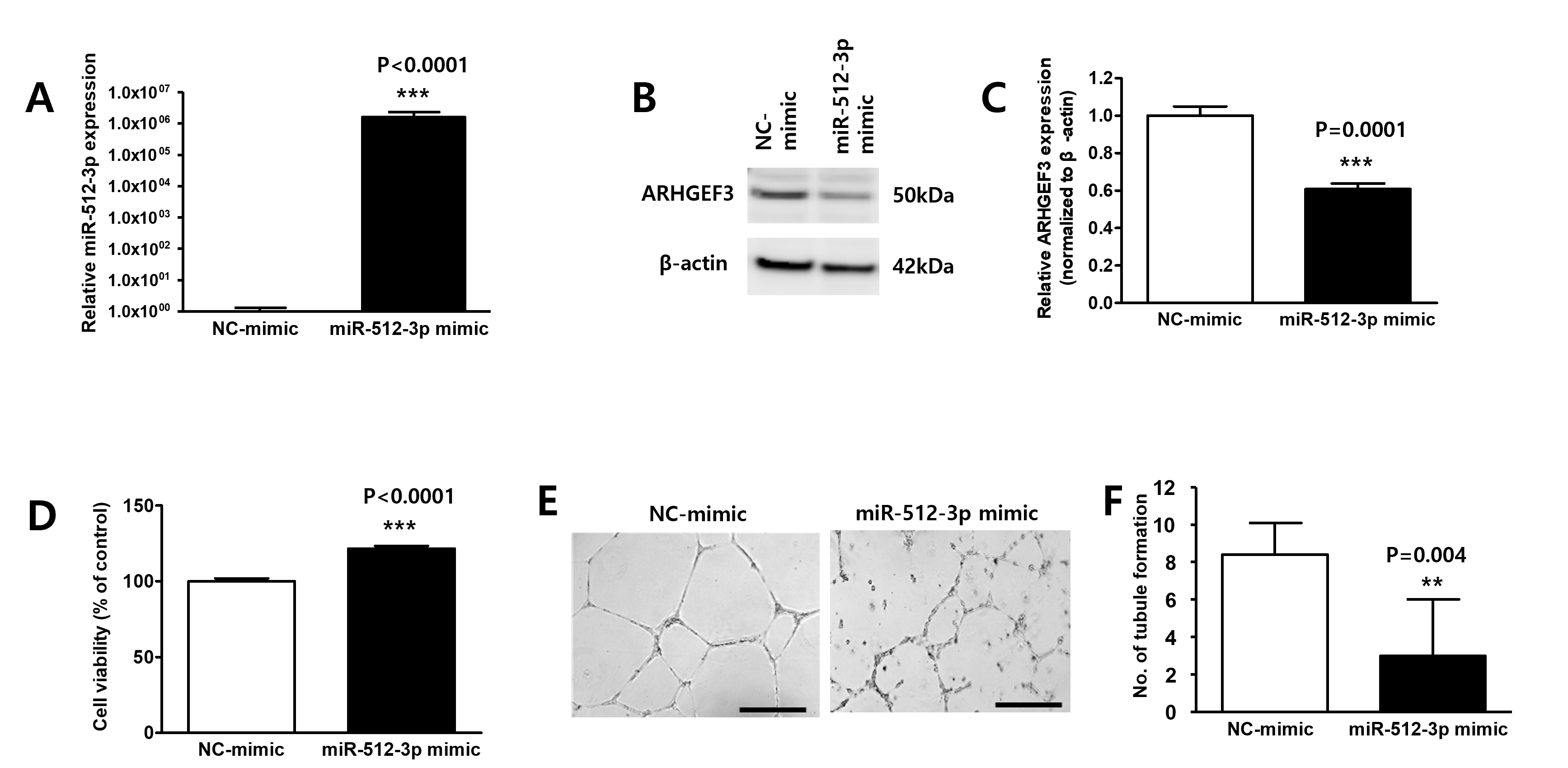

Supplement: Supplementary file 10 — Supplementary Material 10 [file 41598_2025_8796_MOESM10_ESM.tif]
