# Supplementary material for: Plasma extracellular vesicle-associated miR-512-3p modulates angiogenesis in pediatric Moyamoya disease by targeting ARHGEF3
Source: Sci Rep. 2025 Jul 9;15:24655. doi: 10.1038/s41598-025-08796-4 (PMC12241339; doi:10.1038/s41598-025-08796-4)
Supplement: Supplementary file 11 — Supplementary Material 11 [file 41598_2025_8796_MOESM11_ESM.docx]

**Supplementary Figure Legends**

**Supplementary Figure S1. Study workflow**

**Supplementary Figure S2. Expression of membrane proteins in plasma-derived extracellular vesicles (EVs).** (A) Immunoblotting demonstrated flotillin-1 was identified in EVs isolated from control (N=8) and moyamoya (MMD, N=8) plasma, whereas cytochrome C was not detected. The labeling of each sample was based on the patient ID in Supplementary Table 1. (B) The expression of flotillin-1 is greater in EVs isolated from control plasma than in those isolated from MMD patients. (C) The Exo-Check exosome antibody array, containing 12 spots (8 EV-specific markers: CD63, CD81, TSG101, ALIX, FLOT1, EpCAM, ICAM1, ANXA5; and 4 controls: GM130 for cis-Golgi contamination, 2 positive controls, and a blank spot), was used to assess EVs from control (N=1) and MMD (N=1) plasma. Representative figures demonstrate that both groups displayed signals for exosome-specific markers and positive controls (PC), with negligible GM130 contamination.

**Supplementary Figure S3. Differential expression of miRNAs in extracellular vesicles (EVs) from plasma of control individuals and moyamoya disease (MMD) patients.** (A) Unsupervised hierarchical clustering based on NanoString miRNA expression profiles from control (N = 10) and MMD (N = 14) EVs, showing partial group segregation. (B) Expression scatter plots of four downregulated miRNAs (miR-219a-2-3p, miR-3136-5p, miR-1268a, and miR-320e) across control and MMD EVs. (C) Receiver operating characteristic (ROC) curve of four downregulated miRNAs based on NanoString data. (D) Multivariable ROC curve combining miR-512-3p, miR-320e, and miR-1268a.

**Supplementary Figure S4. Differential expression of mRNAs targeted by miRNAs in endothelial colony-forming cells (ECFCs) from patients with moyamoya disease (MMD).** (A) Heatmap depicting the differential gene expression profiles of genes targeted by miRNAs in extracellular vesicles (EVs) from MMD plasma between normal and MMD ECFCs. Green indicates downregulated genes, while red indicates upregulated genes. (B) The most enriched molecular functions of the target genes are unknown.

**Supplementary Figure S5. Sequence complementarity between miR-512-3p and 3′ untranslated region of ARHGEF3**

**Supplementary Figure S6. Biological effects of miR-512-3p overexpression in HUVECs.** (A) HUVECs were transfected with miR-512-3p mimic (50 nM) or a negative control mimic (NC-mimic), followed by molecular and functional analyses. (B) Representative western blot analysis showing a reduction in ARHGEF3 protein levels following miR-512-3p overexpression in HUVECs. β-Actin was used as a loading control for protein normalization. (C) Quantification of ARHGEF3 protein expression normalized to β-Actin, as determined by densitometry. (D) Cell viability was modestly but significantly increased following miR-512-3p overexpression. (E) Representative images of in vitro tubule formation assays demonstrating impaired angiogenic function in HUVECs after miR-512-3p overexpression. Scale bars = 200 μm. (F) Quantitative analysis of tube branch number (mean ± SD) was performed using Student’s t-test. *P < 0.05, **P < 0.001, ***P<0.0001.
